# The Price of Remission: Modeling the Total Financial Burden, Quality-of-Life Impairment, and Healthcare Dependence in Lupus Compared With Chimeric Antigen Receptor (CAR) T-cell Therapy

**DOI:** 10.7759/cureus.106661

**Published:** 2026-04-08

**Authors:** Kareena Kumar, Kenneth Kim

**Affiliations:** 1 Histopathology, La Jolla Institute for Immunology, La Jolla, USA

**Keywords:** cd19 car t-cell, cost of care, cost of healthcare, economic analysis, lupus, quality-of-life (qol), rheumatology care

## Abstract

Systemic lupus erythematosus (SLE) is an autoimmune disease leading to chronic patient impairment, healthcare dependency, side effects from immunosuppressive drugs, and accumulation of medical costs. A single infusion of chimeric antigen receptor (CAR) T-cell therapy (CTCT) is currently expensive, approaching USD 500,000, but the lifetime costs of SLE, including medications, hospitalizations, travel, mental health effects, and lost income, may surpass this figure within years to over a decade, depending on disease severity. This study compares the medical, lifestyle, and quality-of-life financial burdens of SLE against a single CTCT infusion at a reference price of USD 500,000, suggesting that even at current prices, CTCT may be cost-effective for patients living with high to very severe disease activity. Beyond more quantifiable factors, the chance for complete remission and independence from the healthcare system could represent a major improvement in patient quality of life.

## Introduction

Systemic lupus erythematosus (SLE) is a chronic autoimmune disease that affects about 1.5 million people in the United States and at least five million worldwide, with most being women between the ages of 15 and 44 years. Disease activity is often measured using the SLE Disease Activity Index (SLEDAI), which ranges from 0 to 105 as follows: scores of 0 indicate no activity, 1-5 mild, 6-10 moderate, 11-19 high, and ≥20 very high activity [1,2]. For the purposes of this analysis, we use the broader term "disease severity" to encompass not only SLEDAI activity but also cumulative organ damage, treatment burden, and quality-of-life impact. Symptoms vary by severity, from joint pain, fatigue, and skin rashes in mild cases, to kidney failure, heart and lung complications, and cognitive dysfunction in high to very severe disease. Lupus nephritis, one of the most dangerous SLE manifestations, affects about 40% of patients; of these, 10-20% develop end-stage kidney disease, underscoring the need for more effective therapies.

Current treatment options for SLE include hydroxychloroquine, corticosteroids, azathioprine, methotrexate, and biologic therapies such as belimumab, anifrolumab, and rituximab. Except for hydroxychloroquine, most of these drugs are immunosuppressants. While immunosuppressants reduce inflammation and limit SLE flares, adverse effects can include infection, organ toxicity, and mental health effects such as severe anxiety or depression [3]. Even when SLE is controlled, symptoms rarely disappear completely, and quality-of-life can remain significantly impaired [4]. Annual treatment costs vary considerably by regimen; while conventional agents such as hydroxychloroquine are relatively affordable, patients with moderate-to-severe disease requiring biologic or specialty therapies can incur medication costs exceeding USD 30,000 annually in the United States, adding to the long-term financial burden of disease management [5,6].

Chimeric antigen receptor (CAR) T-cell therapy (CTCT) was originally developed for cancer, but its mechanism, genetic modification of T cells to target cell-surface antigens such as CD19 on B cells, has made CTCT an emerging option for treating autoimmune disease [7]. By depleting autoreactive B cells, CTCT can "reset" humoral immunity rather than broadly suppressing immunity. Early clinical evidence, primarily from small case series and phase I trials, has shown early promise as follows: in a five-patient study of refractory SLE, a single CTCT infusion led to the elimination of pathogenic B cells, disappearance of autoantibodies, and remission within three months [7]. Patients remained off medication for months afterward, with only mild, manageable side effects. Larger phase I/II trials are currently ongoing, but regulatory approval for autoimmune indications has not yet been granted in any geography. Although cytokine release syndrome and neurotoxicity remain concerns, these events appeared rare in early reports, which suggests that CTCT may be able to offer patients something current drugs cannot - drug-free remission and relief from the physical, mental, and lifestyle burdens of lifelong SLE. With ongoing advances and cost reductions, CTCT deserves careful consideration as an alternative to current standards of care. The goal of this study was to compare the costs of living with SLE against a single CTCT infusion at a reference cost of USD 500,000 [8,9].

## Materials and methods

To compare the costs of living with SLE against the cost of CTCT, we used a simplified model that highlights the major drivers of expense when living with lupus. Reported disease severity was stratified into inactive, mild, moderate, high, and very severe categories, with lupus nephritis noted separately because of its higher risk of end-stage kidney disease. Both direct medical spending and indirect burdens, such as lost income, travel, and caregiving, were included. For SLE management, costs were drawn from published reports describing direct medical expenses such as hospitalizations, emergency visits, laboratory testing, and medications [2,5,6,10-20]. To reflect the everyday reality of patients, we also factored in non-medical categories like transportation costs for repeated appointments, home modifications, small medical devices, and part-time caregiving. Productivity losses from missed work or reduced ability to earn income were included as well, since these can be significant in severe disease [4].

We built the cost analysis using multiple published reports from longitudinal studies of lupus patients with varying reported lupus disease severities to establish core cost figures for hospital visits, medication costs, costs for physician appointments, and other direct medical costs not otherwise captured in the foregoing categories. We also relied on studies for income-loss figures. Based on the number of appointments, we added conservative transportation costs. Finally, given the increase in mental health impact with increasing lupus severity, we referenced studies citing quantitative and qualitative cost burden associated with patients suffering from mental health issues. The various studies relied on, together with their scope in terms of the patient census, are listed in Table 1 [2,4,6,10,11,13-29].

**Table 1:** Studies examined for cost analysis.

Studies	Study focus	Year	Geography	Patients
Kan et al. [2]	SLE	2013	US	178
Tanaka et al. [4]	SLE	2025	Japan	205
Grabich et al. [10]	SLE	2022	US	154
Arnaud et al. [11]	SLE	2025	France	29,135
Wang et al. [13]	SLE	2023	China	1,778
Bell et al. [5]	SLE	2023	US	11,663
Doria et al. [14]	SLE	2014	Europe	427
Miyazaki et al. [15]	SLE	2020	Japan	4,733
Huang et al. [16]	SLE	2023	US	1,356
Jiang et al. [17]	SLE	2021	US	2,227
Tanaka et al. [18]	SLE	2018	Japan	295
Murimi-Worstell et al. [6]	SLE	2021	US	17,257
Anandarajah et al. [19]	SLE	2017	US	202
Bell et al. [20]	SLE	2022	US	8,952
Cutler et al. [28]	Depression	2022	US	455,082
Konnopka et al. [29]	Anxiety	2020	Germany	27,673

For CTCT, our base case assumed a single USD 500,000 inpatient infusion with the usual components of leukapheresis, genetic modification of the patient's own T cells, reinfusion, and inpatient monitoring [8,9]. Because cytokine release syndrome (CRS) and neurotoxicity are the most concerning side effects, cost ranges for managing these complications were drawn from oncology data and added when applicable [9,21]. At the same time, we note currently active cost-reduction pathways, such as hospital-based manufacturing, off-the-shelf (allogeneic) CTCT, RNA-based therapy, and outsourced production, that will likely make CTCT cheaper in the future [22-24]. Outpatient delivery, which has already reduced costs in cancer settings, was also considered.

Several key assumptions underlie this model. First, the analytic perspective is that of the patient and payer, reflecting out-of-pocket and insurance expenditures rather than broader societal costs. Second, the CTCT comparison assumes a single infusion resulting in durable remission with no retreatment required and no major complications in the base case; complication costs, when included, are treated as a sensitivity range rather than a primary estimate. Third, because source studies span multiple countries and health systems (Table 1), the cost estimates are anchored to US-based studies, with non-US data used to corroborate directional trends; no formal purchasing power parity or inflation adjustment was applied, which is a recognized limitation. Breakeven was estimated by dividing the CTCT reference price (USD 500,000) by the annual cost estimate for each severity level.

To make these comparisons more accessible, we have also published an online hypothetical calculator (lupuscost.lji.org) where users can select a disease severity range, and optionally input an annual income. The calculator then outputs an estimate of SLE-related costs. The calculator is a generic model and should not be used for individual medical or financial advice, but this model helps illustrate the scale of accumulating financial costs. Together, these methods create an informative way to weigh the lifetime costs of SLE against a single round of CTCT.

## Results

When the cost of CTCT is compared with the expenses associated with ongoing systemic lupus erythematosus, the comparison highlights. Inactive or mild disease may cost USD 6,100-12,500 annually, but moderate cases average approximately USD 25,000 per year, and very severe SLE can exceed USD 68,000 annually [2]. Over a decade, this means that a single patient with very severe disease could easily surpass the USD 500,000 mark in medical bills, medications, and lost productivity. Everyday costs like transportation for repeated appointments, small medical devices, or part-time caregiving accumulate quickly.

Based on a review of the prior studies, Table 2 lists estimated costs by disease severity (rounded to USD 100), including both medical and non-medical categories. Costs for patients with very severe SLE cross the reference CTCT cost in approximately seven to eight years. Estimated breakeven timelines vary by severity level as follows: patients with high disease activity reach breakeven in approximately 11 years, and those with moderate disease activity in approximately 20 years. For mild or inactive SLE, CTCT is not cost-effective at current prices within a clinically meaningful timeframe. This simple breakeven estimate shows how a one-time intervention could become cost-effective over time.

**Table 2:** Cost of lupus by reported disease severity.

Disease severity	Other direct medical costs	Hospital visits	Medications	Gas/travel	Appointments	Other	Income loss	Total annual cost
Low	$2,500	$500	$1,200	$300	$400	$200	$1,000	$6,100
Mild	$5,000	$1,000	$2,000	$500	$600	$400	$3,000	$12,500
Moderate	$10,000	$2,500	$4,000	$800	$1,000	$700	$6,000	$25,000
High	$20,000	$4,000	$6,500	$1,200	$1,500	$1,200	$10,000	$44,400
Very high	$30,000	$7,000	$10,000	$2,000	$2,500	$2,000	$15,000	$68,500

Looking forward, emerging strategies, such as off-the-shelf (allogeneic) CTCT, RNA delivery, or hospital-based manufacturing, could reduce CAR T manufacturing costs to USD 80,000 or below [22-24]. If such reductions are realized, the breakeven point would shift dramatically, with a chance for remission becoming not only medically desirable but also financially compelling.

Quality-of-life and mental health burden

The monetary cost of SLE is only one side of the story. Patients with SLE often live with daily fatigue, joint pain, and the unpredictability of flares [5], all of which interfere with work, family life, and even simple routines [25]. Severe SLE can make it difficult to leave the house, and lupus nephritis in particular is associated with dialysis or long hospital stays and complex treatment schedules.

Mental health burdens are also striking. Studies have found that between 20% and 30% of SLE patients meet criteria for depression, with anxiety levels even higher [25-27]. The presence of major depressive disorder has been linked to higher medical costs, greater disability, and a poorer quality-of-life [28,29]. Medications used for lupus, especially corticosteroids and other immunosuppressants, can make these problems worse, sometimes contributing to severe anxiety or suicidal ideation [3,30].

Even if not every dollar can be assigned to a cost category, the takeaway is that quality-of-life losses are real and costly. CTCT, by inducing remission or cure, has the potential to reduce not only direct medical costs but also the less visible tolls of distress, fatigue, and dependence. Opportunity costs, or losses in productivity and quality-of-life due to time and resources spent on suffering from or being treated for SLE, are difficult to measure but should certainly not be ignored in the final analysis.

Risks and safety with CTCT

CTCT is not risk-free. Both CRS and neurotoxicity can be life-threatening if not monitored closely. CRS involves an intense inflammatory response that can affect multiple organ systems. Neurotoxicity may lead to confusion, seizures, or, in rare cases, long-term neurological damage. In oncology, managing these events can add USD 30,000-50,000 to a patient's care. For SLE, data are still limited. The small five-patient trial reported only mild, treatable CRS and no major neurotoxicity events [7]. Still, until larger trials are conducted, there is uncertainty about how frequently these complications will occur and whether the risk profile will look the same as in cancer patients. These considerations are important because a one-time therapy is only cost-effective if the complications are not too frequent or severe. As regimens improve and administration shifts to outpatient or hybrid settings, it is possible that risks will become more predictable and less expensive to manage.

## Discussion

The comparison between lifelong SLE care and a one-time CTCT highlights both the opportunity and the uncertainty of this novel therapy. From a financial perspective, the arithmetic suggests that for patients with very severe SLE, the cumulative costs of medications, hospitalizations, and productivity losses can exceed the upfront cost of CTCT in less than a decade of living with SLE. Even if CTCT remains priced near USD 500,000, the lifetime burden of very severe SLE is high enough that a breakeven point is possible within eight years, at least under the assumptions tested. With future price declines from hospital-based manufacturing, donor cells, RNA-based delivery, or offshore production, the breakeven could arrive even sooner [21-24]. Many of our source publications show data before more recent monetary inflation waves. If the nominal value of CTCT is decreasing or inflating at a slower rate than the costs of living with lupus, then the cost of CTCT may indeed be falling in real terms more than the numbers would indicate. These comparisons are intended to be illustrative and exploratory rather than predictive; individual patient circumstances, insurance coverage, and regional cost structures will differ substantially.

At the same time, clinical evidence supporting the efficacy of CTCT in lupus remains limited. Small clinical trials have produced promising results, with all patients achieving remission and remaining off medication for months, but this cannot yet be generalized to the broader SLE population [7]. Key uncertainties remain about remission durability, the need for retreatment, and the true rate of adverse events like cytokine release syndrome (CRS) or neurotoxicity in autoimmune settings. Larger forthcoming trials will determine whether CTCT can consistently provide safe, drug-free remission.

It is also important to weigh the non-monetary aspects. Patients with SLE face not only direct medical expenses but also daily fatigue, pain, and the mental health burdens of chronic disease [25]. Immunosuppressive drugs can contribute to anxiety, depression, and long-term organ toxicity, which further erode quality-of-life [3,30]. In this context, CTCT represents more than just a financial calculation; it offers the possibility of liberation from symptoms, opportunity costs, and the cycle of dependence on immunosuppression and the healthcare system.

Going forward, the path toward CTCT in SLE will depend on several milestones as follows: larger and longer clinical trials, regulatory approval for autoimmune indications, broader insurance coverage, and continued innovation in therapeutic manufacturing. Even at current prices, the therapy may be cost-effective for severe disease, but CTCT's full potential will only be realized if it becomes safer, more affordable, and more widely accessible. From both a clinical and human perspective, CTCT shows early potential to change the long-term quality-of-life for patients with SLE in ways that current treatments cannot.

Study limitations

We used a simple model to compare the financial burden of living with SLE to the cost of a single dose of CTCT. Our model did not account for all factors, such as inflation, regional cost differences, or changes in insurance coverage. The analytic perspective reflects patient and payer costs rather than broader societal costs; this is a recognized limitation. Productivity loss and caregiving costs were estimated from existing reports, but will vary from person to person. One study examined a top-down population methodology, allocating regional costs on a per capita basis rather than tracking individual patients, and was therefore excluded from per-patient cost calculations; this exclusion is noted as a constraint on the comprehensiveness of the mental health cost estimates [29]. The clinical data for CTCT in lupus are still early, based on small studies. We also do not yet know how long remissions last, how often retreatment might be needed, or what side effects could arise in larger groups. These unknowns could change the breakeven price of CTCT versus current SLE therapies. Our online calculator is meant to give a rough idea of cost burden, not to offer medical or financial advice. More data and thorough analyses will be needed as CTCT trials continue and costs change.

## Conclusions

Systemic lupus erythematosus imposes a cumulative financial burden that, for patients with high to very severe disease activity, can surpass the current cost of a single CTCT infusion within a decade. This exploratory model suggests that CTCT deserves consideration not only as a clinical option but as a potentially cost-effective one for the most severely affected patients. The quality-of-life gains, freedom from chronic immunosuppression, relief from fatigue and pain, and the possibility of drug-free remission, add a dimension to this calculation that numbers alone cannot fully capture. These findings are necessarily illustrative rather than predictive; remission durability, retreatment rates, and real-world complication rates in autoimmune settings remain to be established by larger trials. As the clinical evidence matures and manufacturing costs decline, the case for CTCT in SLE is likely to strengthen on both medical and economic grounds.
